# Viral Determinants of miR-122-Independent Hepatitis C Virus Replication

**DOI:** 10.1128/mSphere.00009-15

**Published:** 2015-11-25

**Authors:** Sharon E. Hopcraft, Kristopher D. Azarm, Benjamin Israelow, Nicolas Lévêque, Megan C. Schwarz, Tien-Huei Hsu, Matthew T. Chambers, Marion Sourisseau, Bert L. Semler, Matthew J. Evans

**Affiliations:** a Department of Microbiology, Icahn School of Medicine at Mount Sinai, New York, New York, USA; b Clinical and Molecular Virology Unit (EA-4684 CardioVir), School of Medicine, University of Reims Champagne-Ardenne, Reims, France; c Department of Microbiology and Molecular Genetics, School of Medicine, University of California Irvine, California, USA; University of Pittsburgh School of Medicine

**Keywords:** hepatitis C virus, microRNA, miR-122, CRISPR

## Abstract

Hepatitis C virus (HCV) replication requires binding of the liver-specific microRNA (miRNA) miR-122 to two sites in the HCV 5′ untranslated region (UTR). Although we and others have shown that viral genetics impact the amount of active miR-122 required for replication, it is unclear if HCV can replicate in the complete absence of this miRNA. To probe the absolute requirements for miR-122 and the genetic basis for those requirements, we used clustered regularly interspaced short palindromic repeat (CRISPR) technology to knock out miR-122 in Huh-7.5 cells and reconstituted these knockout (KO) cells with either wild-type miR-122 or a mutated version of this miRNA. We then characterized the replication of the wild-type virus, as well as a mutated HCV bearing 5′ UTR substitutions to restore binding to the mutated miR-122, in miR-122 KO Huh-7.5 cells expressing no, wild-type, or mutated miR-122. We found that while replication was most efficient when wild-type or mutated HCV was provided with the matched miR-122, inefficient replication could be observed in cells expressing the mismatched miR-122 or no miR-122. We then selected viruses capable of replicating in cells expressing noncognate miR-122 RNAs. Unexpectedly, these viruses contained multiple mutations throughout their first 42 nucleotides that would not be predicted to enhance binding of the provided miR-122. These mutations increased HCV RNA replication in cells expressing either the mismatched miR-122 or no miR-122. These data provide new evidence that HCV replication can occur independently of miR-122 and provide unexpected insights into how HCV genetics influence miR-122 requirements.

**IMPORTANCE** Hepatitis C virus (HCV) is the leading cause of liver cancer in the Western Hemisphere. HCV infection requires miR-122, which is expressed only in liver cells, and thus is one reason that replication of this virus occurs efficiently only in cells of hepatic origin. To understand how HCV genetics impact miR-122 usage, we knocked out miR-122 using clustered regularly interspaced short palindromic repeat (CRISPR) technology and adapted virus to replicate in the presence of noncognate miR-122 RNAs. In doing so, we identified viral mutations that allow replication in the complete absence of miR-122. This work provides new insights into how HCV genetics influence miR-122 requirements and proves that replication can occur without this miRNA, which has broad implications for how HCV tropism is maintained.

## Abstract (precis)

Hepatitis C virus (HCV) is the leading cause of liver cancer in the Western Hemisphere. HCV infection requires miR-122, which is expressed only in liver cells, and thus is one reason that replication of this virus occurs efficiently only in cells of hepatic origin. To understand how HCV genetics impact miR-122 usage, we knocked out miR-122 using clustered regularly interspaced short palindromic repeat (CRISPR) technology and adapted virus to replicate in the presence of noncognate miR-122 RNAs. In doing so, we identified viral mutations that allow replication in the complete absence of miR-122. This work provides new insights into how HCV genetics influence miR-122 requirements and proves that replication can occur without this miRNA, which has broad implications for how HCV tropism is maintained.

## INTRODUCTION

Hepatitis C virus (HCV) is a major human pathogen, with nearly 3% of the world’s population currently infected (1). HCV is hepatotropic and the causative agent of the majority of liver cancers in the Western Hemisphere (2). One required host factor that influences the hepatotropism of HCV is the liver-specific microRNA (miRNA) miR-122. The relationship between miR-122 and HCV is unusual in that, rather than having a repressive effect on translation that would in turn suppress HCV infection, miR-122 increases HCV replication (3). This effect is mediated through the direct interaction of miR-122 and the HCV RNA genome and is not a result of miR-122 modulation of the cellular environment (4, 5). However, the precise mechanism by which miR-122 stimulates replication has yet to be elucidated.

The first 42 nucleotides of the HCV 5′ untranslated region (UTR) contain two miR-122 seed sequence binding sites (3, 4). Mutations in these sites disrupt HCV RNA accumulation, while complementary mutations in miR-122 rescue this phenotype (3, 4, 6–10). miR-122 also forms a secondary contact with the HCV genome through nucleotides near the 3′ end of the miRNA (8, 11). Binding of miR-122 to the 5′ UTR results in a modest increase in translation (6, 7, 12), as well as stabilizes the genome (13) by protecting it from exoribonucleases Xrn1 (14) and Xrn2 (15). Recent evidence also indicates that miR-122 competes with poly(rC)-binding protein 2 (PCBP2) to bind the 5′ UTR and stimulate RNA synthesis (16). However, there may be additional functions of miR-122 in the HCV life cycle that have yet to be described.

HCV genetics influence the quantity of miR-122 required for replication. We have previously identified a single nucleotide change, G28A, between the miR-122 binding sites that improves the ability of the chimeric genotype 2a Jc1 genome to replicate in the presence of inhibitors of miR-122 (17). Furthermore, A28 was determined to be a global determinant of resistance to miR-122 inhibitors, as all isolates containing this nucleotide demonstrated improved replication when active miR-122 is scarce, compared to genomes containing a G at this position. However, HCV genomes with A28 exhibited different degrees of miR-122 inhibitor resistance, which indicated that other viral sequences impact miR-122 usage. Moreover, we could not discern if these genomes were replicating entirely independently of miR-122, given that such inhibitors are unlikely to inhibit all miR-122.

The goal of this study was to take an unbiased approach to probe how HCV genetics influence miR-122 usage in cells that completely lack miR-122. Toward that end, we generated a Huh-7.5 miR-122 knockout (KO) cell line; complemented those cells with no miR-122, wild-type miR-122, or mutated miR-122 RNAs; and characterized the replication of HCV bearing either a wild-type or a mutated 5′ UTR in those cells. We then selected viruses capable of replicating in the presence of noncognate miR-122s and, in doing so, identified mutations that allow miR-122-independent replication. These data provide us with a deeper understanding of the genetic basis for miR-122 requirements.

## RESULTS

### Characterization of HCV replication in miR-122 KO Huh-7.5 cells.

**FIG 1:**
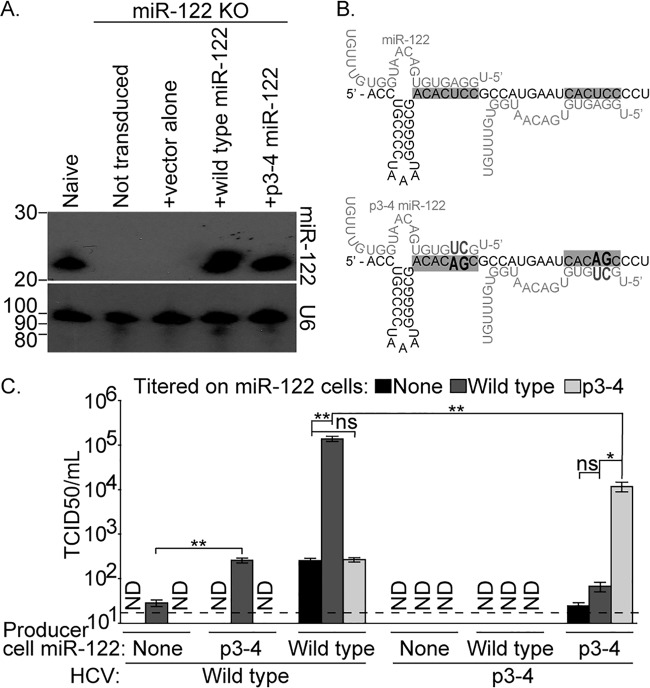
Characterization of a miR-122 KO Huh-7.5 cell clone. (A) Northern blot analysis for miR-122 or U6 was performed with total RNA isolated from naive Huh-7.5 cells or a miR-122 KO Huh-7.5 cell clone either not expressing a transgene (Not transduced) or transduced with lentiviral particles to express the vector alone, wild-type miR-122, or p3-4 miR-122. Approximate nucleotide sequence sizes (in bases) are indicated to the left of each blot. (B) Illustrations of wild-type (top) or p3-4 (bottom) Jc1 5′ UTRs in black text showing the miR-122 seed sequence binding sites (gray boxes) in complex with wild-type or p3-4 miR-122 molecules in gray text. The p3-4 mutations are in bold, enlarged letters. (C) The relative titers of wild-type or p3-4 Jc1 virus produced in cells expressing the indicated miR-122 were determined by limiting-dilution assay on miR-122 KO Huh-7.5 cells expressing no, wild-type, or p3-4 miR-122. Values represent means and standard errors of three independent assays of tissue culture infective doses (TCID_50_) and are expressed in TCID_50_ per milliliter (TCID_50_/ml). The limit of detection is indicated by the dashed line. ND, not detected. ns, not significant; *, *P* < 0.05; **, *P* < 0.01 (Student’s *t* test).

Huh-7.5 is a line of human hepatocellular carcinoma-derived cells that are highly permissive to HCV infection and express miR-122 (18). We generated a miR-122 KO Huh-7.5 single-cell clone using clustered regularly interspaced short palindromic repeat(s) (CRISPR) technology (19–21). miR-122 expression in this clone was undetectable by Northern blot analysis (Fig. 1A). We then transduced these miR-122 KO cells to stably express wild-type miR-122; a mutated miR-122 in which the third and fourth positions of the miRNA are altered, referred to as p3-4 miR-122 (Fig. 1B); or the vector alone. p3-4 miR-122 does not bind wild-type HCV RNA and thus does not support wild-type HCV RNA accumulation, but RNA replication is restored by complementary mutations in both miR-122 binding sites in the HCV 5′ UTR, termed p3-4 HCV (3, 4, 9). Following transduction, Northern blot analysis showed that both lentivirus-derived wild-type and p3-4 miR-122 RNAs were expressed at levels similar to each other in each cell population and endogenous miR-122 in naive Huh-7.5 cells (Fig. 1A).

To determine the ability of these cell populations to support HCV replication and to produce infectious virus, we transfected each cell line with either wild-type or p3-4 HCV RNA transcribed from a genotype 2a chimeric HCV genome bearing the 5′ UTR and structural proteins of the HC-J6 isolate and the nonstructural proteins from JFH-1, referred to as Jc1 (22). The titers of infectious virus in the supernatant of transfected cells were then determined on cells expressing no, wild-type, or p3-4 miR-122. Infectious wild-type Jc1 HCV was produced by transfection of all three cell lines, even those without any miR-122, indicating that some miRNA-independent replication can occur (Fig. 1C, first three sets of three columns). However, infectious wild-type virus made in cells expressing no miR-122 or p3-4 miR-122 could be detected only in cells expressing wild-type miR-122 (Fig. 1C, middle column of first two data sets). Wild-type virus produced in p3-4 miR-122-expressing cells grew to 9-fold higher titers than virus produced in cells expressing no miR-122, indicating that wild-type virus replication was improved slightly by the presence of a mismatched miR-122. On the other hand, wild-type virus produced in wild-type miR-122-expressing cells was equally infectious in cells expressing no miR-122 and cells expressing p3-4 miR-122, again indicating the capacity for miRNA-independent replication, but suggesting that the benefit of the mutated miRNA for wild-type virus is not consistent. However, populations of cells expressing no miR-122 and cells expressing p3-4 miR-122 were both at least 520-fold less susceptible to infection with this virus than cells expressing wild-type miR-122.

No infectious p3-4 HCV was detectable in cells expressing no, wild-type, or p3-4 miR-122 following the transfection of each cell population with the p3-4 viral RNA (Fig. 1C, fourth and fifth data sets). On the other hand, a stock of p3-4 virus made in p3-4 miR-122-expressing cells was also able to infect cells expressing no or wild-type miR-122. Finally, the stock of p3-4 virus grew to high titers in cells expressing p3-4 miR-122, to a level 11.8-fold lower than the stock of wild-type virus in wild-type miR-122-expressing cells, indicating that altering HCV miR-122 usage has only a moderate impact on viral fitness.

### Adaptation of HCV to replicate using noncognate miR-122 RNAs.

**FIG 2:**
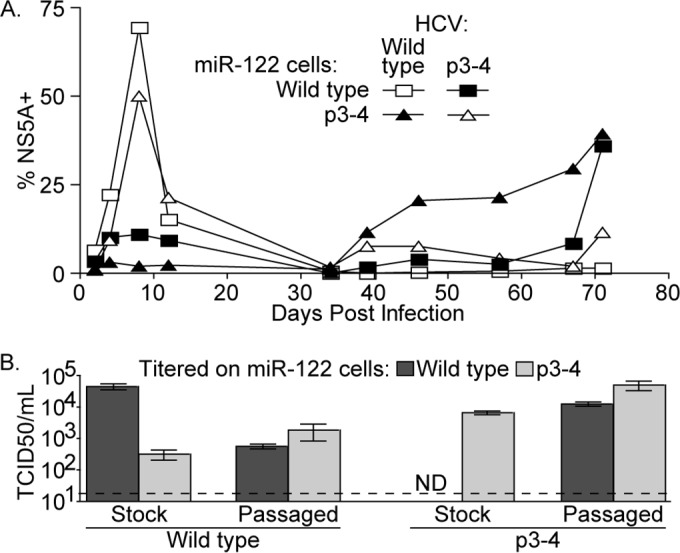
Adaptation of HCV to replicate using noncognate miR-122 RNAs. (A) HCV persistence in serially passaged wild-type or p3-4 Jc1 HCV-infected miR-122 KO cells expressing either wild-type or p3-4 miR-122 was monitored on the postinfection days indicated by HCV NS5A staining and FACS analysis. Sets in which the virus is provided with a matched miRNA are indicated by open symbols, and those in which the virus is provided with a mismatched miRNA are indicated by filled symbols. (B) The relative titers of stocks of wild-type or p3-4 Jc1, as reference points, and supernatants containing virus passaged in cells expressing the mismatched miRNA were determined by limiting-dilution assay on miR-122 KO Huh-7.5 cells expressing either wild-type (dark gray) or p3-4 (light gray) miR-122. TCID_50_/ml means and standard errors of three independent assays are shown. The limit of detection is indicated by the dashed line. ND, not detected.

To select viruses capable of replicating in the presence of a mismatched miRNA, we infected wild-type or p3-4 miR-122-expressing cells with stocks of wild-type and p3-4 HCV. All four infections were initiated at the same multiplicity of infection (MOI), and we used the ability of each virus to spread in the culture, indicated by staining of cells for the HCV protein NS5A, as a gauge of how efficiently each virus was using the provided miRNA for replication. Wild-type and p3-4 viruses spread rapidly through cells expressing complementary miR-122s, infecting 70 and 50% of the cells by 8 days postinfection, respectively (Fig. 2A, open symbols). Infection in both of these cultures then waned, likely reflecting the cytotoxicity of HCV in culture, which would result in selection for cells that are not HCV susceptible. In contrast, wild-type and p3-4 HCV did not spread efficiently among cells expressing noncognate miR-122s. At 34 days postinfection, uninfected wild-type or p3-4 miR-122-expressing cells were mixed with infected wild-type or p3-4 miR-122-expressing cells, respectively, to amplify any remaining virus in these populations. Infection of p3-4 miR-122-expressing cells by wild-type HCV gradually increased, ultimately reaching 39% of the cells at the end of the experiment, while infection of wild-type miR-122-expressing cells by p3-4 virus remained low until the final time point, when 36% of the cells were infected (Fig. 2A, filled symbols). This suggested that the passaged viruses had acquired the capacity to replicate efficiently in cells expressing the mismatched miR-122.

To assess if selection of viruses with altered miR-122 requirements had occurred, the infectious virus titers in supernatants from these cultures were determined on miR-122 KO cells expressing either wild-type or p3-4 miR-122. For comparison, stocks of wild-type and p3-4 HCV were assayed in parallel on both cell lines. While the stock of wild-type HCV was 140-fold more infectious in wild-type miR-122-expressing cells than in p3-4 miR-122-expressing cells, wild-type virus passaged in p3-4 miR-122-expressing cells was 3.3-fold more infectious in p3-4 miR-122-expressing cells than in wild-type miR-122-expressing cells (Fig. 2B). Similarly, while the stock of p3-4 HCV was at least 250-fold more infectious in p3-4 miR-122-expressing cells than in wild-type miR-122-expressing cells, p3-4 virus passaged in wild-type miR-122-expressing cells was only 3.9-fold more infectious in p3-4 miR-122-expressing cells. This indicated that both the wild-type and p3-4 viruses passaged in the presence of a mismatched miR-122 adapted to replicate in cells expressing the mismatched miRNA.

### Adapted viruses replicate in cells expressing mismatched miR-122 RNAs.

**FIG 3:**
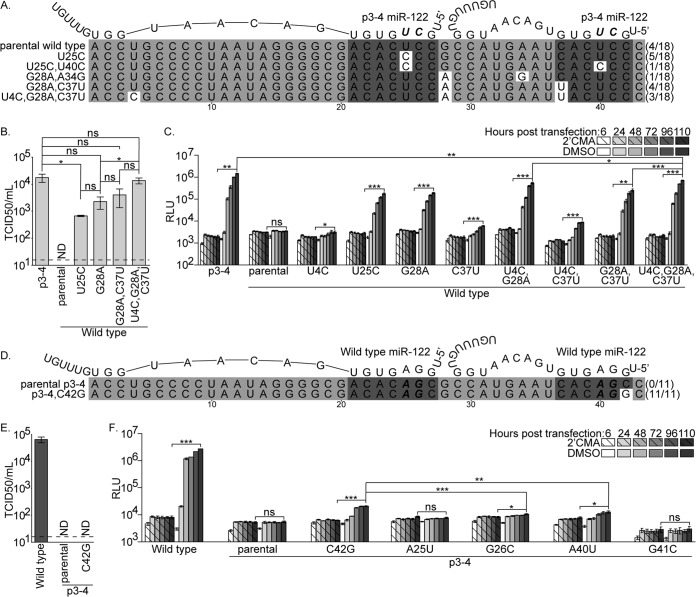
Adapted viruses replicate in mismatched miR-122-expressing cells. (A) 5′ RACE and sequencing was performed with wild-type Jc1 passaged in p3-4 miR-122-expressing cells. Shown is an alignment of the first 43 nucleotides of Jc1 with both miR-122 seed sequence binding sites in dark gray boxes, the identified mutations in white boxes, and nucleotide positions indicated below. The number of times a sequence was identified out of the total number of sequences examined is indicated to the right of each sequence. p3-4 miR-122 molecules are shown above the alignment, with the p3-4 mutations in bold italics. (B) Relative titers of p3-4, parental wild-type, and adapted wild-type Jc1 viruses produced in p3-4 miR-122-expressing miR-122 KO Huh-7.5 cells were determined by limiting-dilution assay on these cells. Shown are means and standard errors of infections with three independent preparations of each virus. The limit of detection is indicated by the dashed line. ND, not detected. (C) Luciferase production was monitored at the indicated times posttransfection of p3-4 miR-122-expressing miR-122 KO Huh-7.5 cells with the p3-4, parental wild-type, or adapted wild-type Jc1 reporter genome. Transfected cells were cultured in the presence of the HCV polymerase inhibitor 2′CMA (hashed bars) or the dimethyl sulfoxide (DMSO) vehicle (solid bars). Shown are representative values from two independent experiments performed in triplicate, and luciferase values are in relative light units (RLU). (D) 5′ RACE and sequencing of p3-4 Jc1 passaged in miR-122 KO Huh-7.5 cells expressing wild-type miR-122 was performed as described for panel A. (E) Relative titers of wild-type, parental p3-4, or adapted p3-4 Jc1 virus produced in wild-type miR-122-expressing miR-122 KO Huh-7.5 cells were determined as described for panel B, and titers were determined on the same cells. (F) Luciferase production was monitored as described for panel C after transfection of wild-type miR-122-expressing miR-122 KO Huh-7.5 cells with wild-type, parental p3-4, and adapted p3-4 monocistronic reporter genomes. ns, not significant; *, *P* < 0.05; **, *P* < 0.01; ***, *P* < 0.001 (Student’s *t* test).

To examine the 5′ UTRs of the passaged viruses, we performed 5′ rapid amplification of cDNA ends (5′ RACE) on viral RNA and sequenced the resulting products. For the wild-type virus that was passaged in p3-4 miR-122-expressing cells, we observed a mixture of wild-type and mutated viruses. The sequences identified and their frequencies are depicted in Fig. 3A. We found viral RNAs containing a single U25C mutation or U25C and U40C. While the U25C and U40C mutations fall within the first and second miR-122 binding sites, respectively, they would not be predicted to enhance binding to p3-4 miR-122. Additionally, several viral RNAs were identified containing the previously described G28A mutation, including G28A in combination with U4C, A34G, and C37U. Of these mutations, only C37U falls within a miR-122 binding site and would not be predicted to enhance the binding of p3-4 miR-122. Importantly, none of these mutations was identified in wild-type virus that was cultured in wild-type miR-122-expressing cells (data not shown).

To assess the impact of the identified mutations on wild-type virus replication in p3-4 miR-122-expressing cells, these mutations were cloned into the parental Jc1 HCV genome. Virus was produced in these cells, and titers were determined on the same cells. As observed above, in p3-4 miR-122-expressing cells, while p3-4 virus grew to high titers, no infectious wild-type virus was detected (Fig. 3B). With the limit of detection of this assay, this result indicated that wild-type HCV was at least 650-fold less infectious than p3-4 HCV in these cells. In contrast, HCV with wild-type miR-122 binding sites and a U25C or G28A single-nucleotide change was infectious in these cells, at a level 26- and 7.7-fold lower than the p3-4 virus level, respectively. Combining G28A with C37U slightly improved virus titers in these cells, and the addition of a third change, U4C, to G28A and C37U enhanced virus titers to a level equal to that of the p3-4 virus. This confirmed that the 5′ UTR mutations identified in wild-type virus passaged in p3-4 miR-122-expressing cells had a positive impact on viral replication in these cells.

The sensitivity of the above-described assay was likely compromised by the immunostaining detection method and the requirement for each viral genome to replicate in both the initially transfected cells and the cells used to determine viral titers. To more sensitively compare viral replication fitness, we next directly examined replication in the transfected cell populations by using viral genomes that encoded the gene for *Gaussia* luciferase (GLuc) between the HCV genes for p7 and NS2 (23). In cells, GLuc expressed from these reporter genomes is secreted into the culture supernatant, which we assayed at numerous time points posttransfection. HCV RNA replication was gauged as an increase in GLuc secretion over time in comparison with that in cells incubated with the HCV polymerase inhibitor 2′-C-methyladenosine (2′CMA) (24). While the p3-4 virus replicated efficiently in p3-4 miR-122-expressing cells, HCV with wild-type miR-122 binding sites did not replicate in these cells (Fig. 3C). However, the U4C and C37U mutations individually promoted detectable RNA replication to 1.6- and 3.2-fold over the 2′CMA background, respectively. These two mutations combined had a larger impact than either mutation alone, increasing replication to 7-fold over the background. On the other hand, virus with wild-type miR-122 binding sites and the U25C or G28A mutation replicated at levels 61- or 54-fold over the background, respectively. Adding either U4C or C37U in combination with G28A improved replication, while incorporating all three mutations had the greatest impact on replication, to only 2.1-fold lower than that of the p3-4 virus in these cells. These data demonstrate that the U4C, U25C, G28A, and C37U mutations each have an impact on replication and confirm that the most fit of the identified adapted wild-type miR-122 binding site genomes contains U4C, G28A, and C37U.

Next we examined the 5′ UTR sequences of the p3-4 miR-122 binding site virus passaged in the presence of wild-type miR-122. All of the 11 passaged viruses sequenced maintained the original p3-4 mutations and contained a single additional change of C42G (Fig. 3D). Although this mutation falls at the end of the second miR-122 binding site, it would not be predicted to enhance binding to wild-type miR-122. The C42G mutation did not arise when the p3-4 virus was cultured in p3-4 miR-122-expressing cells (data not shown). To test if the identified C42G mutation had an impact on the replication of p3-4 miR-122 binding site HCV in the presence of wild-type miR-122, this mutation was cloned into the parental p3-4 virus. Virus was then produced in wild-type miR-122-expressing cells, and titers were determined on the same cells. While wild-type HCV produced in wild-type miR-122-expressing cells grew to high titers in those cells, no infectious p3-4 or p3-4,C42G virus was detected (Fig. 3E). To examine replication in a more sensitive assay, we transfected wild-type miR-122-expressing cells with reporter virus genomes and found that, as expected, wild-type HCV replicated efficiently in wild-type miR-122-expressing cells, while p3-4 HCV did not replicate in these cells (Fig. 3F). However, p3-4,C42G HCV did replicate in the presence of wild-type miR-122 to a level 3.4-fold over the background, indicating that the C42G mutation does indeed enhance p3-4 HCV fitness in the presence of a mismatched miR-122.

We hypothesized that the p3-4 mutations did not revert to wild-type sequences while cultured in the presence of wild-type miR-122 because individual changes to wild-type sequences would not confer a fitness advantage. To test this hypothesis, we cloned p3-4 reporter genomes in which each of the four p3-4 mutations was reverted individually to the wild-type sequence. Two of the four p3-4 single-nucleotide revertants, A25U and G41C, did not replicate detectably in wild-type miR-122-expressing cells (Fig. 3F). On the other hand, when G26 or A40 was reverted back to wild-type C26 or U40, the virus replicated, albeit inefficiently, at a level only 1.3- or 1.6-fold over the background, respectively. While these reversions did confer a modest fitness advantage over the p3-4 miR-122 binding site virus in wild-type miR-122-expressing cells, these viruses did not replicate as well as the p3-4,C42G virus, providing a possible explanation for why C42G, but not wild-type sequences, was selected.

### Adapted viruses replicate in miR-122 KO cells.

**FIG 4:**
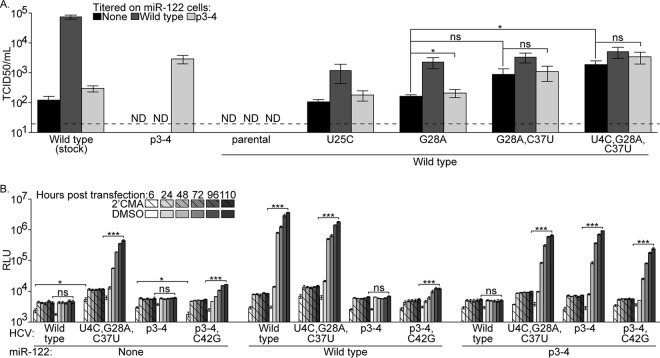
Adapted viruses replicate in miR-122 KO cells. (A) Relative titers of p3-4, parental wild-type, and adapted wild-type Jc1 viruses produced in p3-4 miR-122-expressing miR-122 KO Huh-7.5 cells or a stock of wild-type Jc1 were determined by limiting-dilution assays on miR-122 KO Huh-7.5 cells expressing no, wild-type, or p3-4 miR-122. Shown are means and standard errors of two independent infections with three independent preparations of each virus. The limit of detection is indicated by the dashed line. (B) Luciferase production was monitored at the indicated times posttransfection of miR-122 KO Huh-7.5 cells expressing no, wild-type, or p3-4 miR-122 with parental wild-type, adapted wild-type, parental p3-4, and adapted p3-4 Jc1 reporter genomes. Transfected cells were cultured in the presence of the HCV polymerase inhibitor 2′CMA (hashed bars) or the dimethyl sulfoxide (DMSO) vehicle (solid bars). Shown are representative values from two independent experiments performed in triplicate, and luciferase values are in relative light units (RLU). ND, not detected. ns, not significant; *, *P* < 0.05; ***, *P* < 0.001 (Student’s *t* test).

To evaluate the absolute miR-122 requirements of wild-type miR-122 binding site viruses with adaptive mutations, the titers of viruses produced in p3-4 miR-122-expressing cells were determined on miR-122 KO cells expressing no, wild-type, or p3-4 miR-122. For comparison, a stock of wild-type virus was also assayed in these cell types in parallel. While infectious wild-type virus produced in p3-4 miR-122-expressing cells was not detected in any cell type, virus bearing the U25C or G28A mutation alone was infectious in all three cell types, although it grew to the highest titers in wild-type miR-122-expressing cells (Fig. 4A). The addition of C37U or U4C and C37U to the G28A virus did not have a large impact on virus titers in wild-type miR-122-expressing cells but did increase infection of cells expressing no miR-122, where these viruses were 5.4- and 11.4-fold more infectious, respectively, than virus bearing the G28A mutation alone. Furthermore, while a stock of wild-type virus was 602-fold more infectious in wild-type miR-122-expressing cells than in cells expressing no miR-122, the U4C,G28A,C37U wild-type miR-122 binding site virus was only 2.7-fold more infectious in wild-type miR-122-expressing cells, indicating that efficient miR-122-independent replication was occurring.

To examine the efficiency of miR-122-independent replication of adapted viruses in a more sensitive assay, we transfected miR-122 KO cells expressing no, wild-type, or p3-4 miR-122 with reporter genomes bearing parental or adapted wild-type or p3-4 miR-122 binding site 5′ UTRs. Both parental and adapted genomes replicated most efficiently when provided with the complementary miR-122 (Fig. 4B). However, while the parental genomes did not replicate in miR-122 KO cells, both the adapted wild-type (U4C,G28A,C37U) and adapted p3-4 (p3-4,C42G) genomes did replicate, at levels 38- and 3.1-fold over the 2′CMA background, respectively. Remarkably, the replication of the adapted wild-type miR-122 binding site genome in miR-122 KO cells was only 8.1-fold lower than the replication of the parental wild-type genome in wild-type miR-122-expressing cells. The replication of the adapted p3-4 genome was similar in cells expressing wild-type or no miR-122, while the replication of the adapted wild-type miR-122 binding site genome was 1.7-fold higher in cells expressing p3-4 than in cells expressing no miR-122, suggesting that the mismatched miRNA still had an impact on the replication of this virus. Additionally, transfection of cells expressing no miR-122 with the U4C,G28A,C37U wild-type miR-122 binding site genome resulted in a 2.6-fold increase in the luciferase signal at the earliest time point compared to transfection with the parental wild-type miR-122 binding site genome. On the other hand, transfection of these cells with the adapted p3-4,C42G genome resulted in a 1.3-fold decrease in the signal at the earliest time point compared to transfection with the parental p3-4 miR-122 binding site genome, indicating that the adaptive mutations identified impacted the quantity of luciferase produced from the translation of input HCV RNA.

## DISCUSSION

The goals of this study were to determine the absolute requirements of miR-122 for HCV replication and to probe how HCV genetics modulate this relationship. Toward these ends, we established a Huh-7.5 miR-122 KO single-cell clone, which was complemented with either wild-type or mutated miR-122 RNA. Not surprisingly, wild-type and mutated HCV replicated most efficiently when produced in and assayed on cells expressing the complementary miR-122. However, even in cells expressing p3-4 miR-122, p3-4 HCV was 1 log less fit than wild-type HCV in wild-type miR-122-expressing cells. One possible explanation for this difference in replication is that the p3-4 mutations disrupt structures in the 3′ UTR of the negative strand of the HCV genome that are important for promoting the synthesis of positive-sense HCV RNA. Alternatively, the p3-4 mutations may impact replication by altering the recruitment of host cell RNA-binding proteins, such as PCBP2 (16, 25–28), to the 5′ UTR.

Stocks of wild-type and p3-4 HCV replicated inefficiently in cells expressing the mismatched miRNA. Long-term culture of viruses in the presence of noncognate miR-122 RNAs resulted in an adaptation that allowed more efficient replication under these conditions. However, these adaptive mutations are not predicted to restore miRNA binding, as one might expect. For instance, the miR-122 binding sites of the p3-4 virus did not revert back to the wild-type sequence in wild-type miR-122-expressing cells. We tested the fitness of single-nucleotide reversions of the p3-4 mutations back to wild-type sequences, and only two of four reversions detectably enhanced fitness. Furthermore, neither of these reversions had as great an impact as the C42G mutation we identified in the passaged p3-4 virus, although this virus still did not exhibit high levels of replication. Thus, efficient replication of the p3-4 HCV in wild-type miR-122-expressing cells may require combinations of adaptive mutations. Given that spread of p3-4 virus in wild-type miR-122-expressing cells was detectable only at the final time point collected, it is possible that we captured only early events in adaptation and that with continued passage, revertants to wild-type sequences or additional mutations to enhance fitness would have been selected.

Similarly, when wild-type virus was adapted to replicate in p3-4 miR-122-expressing cells, mutations to establish binding to p3-4 miR-122 were not selected. Instead, nearly half of the sequenced isolates contained the G28A mutation in combination with other changes. We previously showed that this mutation enhanced the ability of Jc1 genotype 2a HCV to replicate in the presence of miR-122 inhibitors (17). We proposed the model that G28A acted to promote accessibility or binding of miR-122 to the 5′ UTR, which may allow HCV replication even when active miR-122 is scarce. While that remains a reasonable model in cells that express wild-type miR-122, the G28A mutation should not increase p3-4 miR-122 recruitment because this miRNA cannot bind the wild-type 5′ UTR sequence. Rather, in this study we found that G28A, in combination with U4C and C37U, actually promoted miR-122-independent replication. Furthermore, we previously showed that all HCV isolates that exhibited miR-122 inhibitor resistance contained an A at nucleotide 28; however, the degree of resistance varied between A28-bearing HCV isolates (17). Here we show that other 5′ UTR changes impact HCV miR-122 usage. For instance, the genotype 5a SA13 isolate contains both C4 and A28, and this isolate demonstrated the greatest resistance to miR-122 inhibitors of the genomes tested.

While the adapted wild-type miR-122 binding site virus we selected benefitted from both cognate and noncognate miR-122 RNAs, this adapted genome could replicate in miR-122 KO cells. Therefore, these mutations either act by directing HCV to use a miRNA other than miR-122 or alleviate the need to bind a miRNA altogether. Even though some of the identified mutations do not fall within the miR-122 binding sites, they may affect miRNA binding indirectly. Alternatively, these mutations may act by disrupting an activity that miR-122 normally counteracts. Masaki et al. recently showed that miR-122 enhances HCV replication, at least in part, by displacing the translation-promoting factor PCBP2 (16). The adaptive mutations identified above could promote miR-122 independence by reducing the binding of PCBP2 to the 5′ UTR, thereby restoring the balance between RNA synthesis and translation. However, we have been unable to detect a difference in PCBP2 binding to wild-type or U4C,G28A,C37U 5′ UTR RNA by electrophoretic mobility shift, UV cross-linking, or RNA pulldown assays (data not shown). Alternatively, the adaptive mutations may decrease viral RNA targeting by exonucleases, a function that miR-122 has also been shown to perform (14, 15). We did not discern a strong impact of these sequence changes on reporter expression from transfected HCV RNA. However, although such an assay has previously been used to examine viral genome stability, this approach may be complicated by the combined impact of both RNA stability and translation on the reporter signal. Ultimately, the genomes we selected that are capable of miR-122-independent replication may be useful for defining important roles for miR-122 in the HCV life cycle.

Our study provides the strongest evidence to date that HCV can evolve to replicate in the absence of miR-122. This has important implications for our understanding of how the liver tropism of this virus is controlled and whether other cell types in a patient can support infection. Furthermore, our results highlight potential issues with the possible use of miR-122-based drugs for the treatment of HCV infection.

## MATERIALS AND METHODS

### Plasmid construction.

To perform CRISPR-mediated gene KO, expression plasmids encoding a U6 promoter-driven miR-122-specific guide RNA were generated as previously described (29), by using miR-122 target sequence-specific forward and reverse oligonucleotides ME-O-1284 (5′ AGAGCTGTGGAGTGTGACAAGTTTTAGAGCTAGAAATA) and ME-O-1285 (5′ TTGTCACACTCCACAGCTCTCGGTGTTTCGTCCTTTCC).

To generate nonreporter Jc1 HCV plasmids with 5′ UTR mutations, the HCV 5′ UTR through the E1 gene was amplified as an EcoRI-BsiWI fragment by using forward oligonucleotides incorporating specific mutations (sequences are available on request) and reverse oligonucleotide ME-O-1069 (5′ CAACAGTATGCGTACGCGCGTCCAC). The resulting PCR product was cloned into the EcoRI and BsiWI sites of Jc1. To generate the GLuc-expressing Jc1 HCV, the mutant 5′ UTRs were cloned as an EcoRI-BsiWI fragment into the reporter virus.

All PCR-amplified sequences and cloning junctions were verified by sequence analysis.

### Cell culture and cell lines.

293T and Huh-7.5 cells (provided by Charles Rice, Rockefeller University) (18) were grown in Dulbecco’s modified Eagle’s medium (Sigma) with 100 U/ml penicillin, 100 mg/ml streptomycin (Corning Life Sciences), and 5% fetal bovine serum (Gibco BRL Life Technologies). To stably express wild-type and p3-4 miR-122, miR-122 KO Huh-7.5 cells were transduced with TRIP lentiviral vectors encoding puromycin resistance linked to the miR-122 genomic locus as previously described (9) and selected with 1 mg/ml puromycin (Corning Life Sciences).

### CRISPR-mediated gene KO.

Huh-7.5 cells were transduced to express a miR-122 sensor encompassing a transcript encoding green fluorescent protein and five 3′ UTR miR-122 target sites (30). Sensor-expressing cells were then transiently transfected with expression plasmids encoding a human codon-optimized Cas9 protein from *Streptococcus pyogenes* (provided by George Church, Harvard University; Addgene plasmid number 41815) (19) and a miR-122-specific guide RNA targeting base 11 relative to the 5′ end of the mature miRNA (5′ AGAGCTGTGGAGTGTGACAA). Following passage for 2 weeks to allow the turnover of previously produced miR-122 RNAs, derepression of the reporter occurred in about 3% of the transfected cells, which were sorted with a fluorescence-activated cell sorter (FACS), and single-cell clones were derived by dilution cloning.

### Northern blot analysis.

Northern blot analysis was performed as previously described, with equal quantities of RNA and U6 RNA as a loading control (9). A probe (5′ CAAACACCATTGTCACAC) capable of binding to both wild-type and p3-4 miR-122 was used to determine the respective levels in each cell line.

### Virus generation and infection.

Lentivirus production and infection were performed as previously described (31). Nonreporter and GLuc-expressing Jc1 genotype 2a chimeric HCV plasmids were provided by Charles Rice, Rockefeller University (23). HCV was produced and titers were determined by limiting-dilution assay as previously described (32, 33). Staining was performed with the clone 9E10 anti-NS5A antibody (provided by Charles Rice, Rockefeller University) and a goat anti-mouse peroxidase-conjugated antibody (Invitrogen).

HCV infections for adapted virus selection were initiated at an MOI of 0.001. HCV RNA replication was tested by transfection with 3 µg of *in vitro*-transcribed RNA as previously described (34). 2′CMA (24) was provided by Timothy Tellinghuisen (Scripps Research Institute). Luciferase in culture supernatants was measured as previously reported (31).

### Virus sequencing.

RNA was extracted from infected cells with Trizol (Ambion) in accordance with the manufacturer’s protocol, and 500 ng of RNA was used in the 5′ RACE System for Rapid Amplification of cDNA Ends kit (Invitrogen) in accordance with the manufacturer’s protocol. First-strand cDNA synthesis and subsequent PCR were performed with the respective oligonucleotides GSP1 (ME-O-1072; 5′ TGGTGATGCAGGACAGCAGG) and GSP2 (ME-O-836; 5′ CCGCCCGGAAACTTAACGTCTTGT). Two independent PCRs were performed with each cDNA. The PCR products obtained were then cloned and sequenced.

### Statistical analysis.

The statistical significance of differences was determined with an unpaired Student *t* test on Prism software (GraphPad Software). A *P* value of ≤0.05 was considered significant. The values in graphs are means and standard errors of representative experiments performed independently two or three times in triplicate.
